# Synergistic Production of Lycopene and β-Alanine Through Engineered Redox Balancing in *Escherichia coli*

**DOI:** 10.3390/ijms26146727

**Published:** 2025-07-14

**Authors:** Xuanlin Wang, Yingchun Miao, Weifeng Liu, Yong Tao

**Affiliations:** 1 CAS Key Laboratory of Microbial Physiological and Metabolic Engineering, State Key Laboratory of Microbial Resources, Institute of Microbiology, Chinese Academy of Sciences, Beijing 100101, China; 2 College of Life Science, University of Chinese Academy of Sciences, Beijing 100049, China

**Keywords:** lycopene, β-alanine, redox balance, metabolic engineering, *Escherichia coli*

## Abstract

The production of β-alanine from fatty acid feedstocks presents a promising synthetic strategy due to its high carbon yield. However, the excessive reducing power generated during fatty acid utilization disrupts cellular redox balance, adversely affecting metabolism and limiting the efficiency and final yield of β-alanine production. To address this challenge, we engineered a co-production system in which excess reducing equivalents generated during fatty acid β-oxidation and β-alanine biosynthesis were consumed by growth-coupled lycopene biosynthesis. The resulting dual-pathway strain, SA01, achieved 44.78 g/L β-alanine and 3.07 g/L lycopene in bioreactor fermentation, representing a 21.45% increase in β-alanine production compared to the β-alanine-producing strain WA01, and a 74.43% increase in lycopene production compared to the lycopene-producing strain LA01. Further optimization in strain SA06, involving cofactor engineering to shift redox flow from NADH to NADPH, enhanced the titers to 52.78 g/L β-alanine and 3.61 g/L lycopene. Metabolite analysis confirmed a decrease in intracellular NADH and FADH_2_ levels in SA06, indicating restoration of redox balance during the late fermentation phase. Additional improvements in the fermentation process, including gradual carbon source switching, optimization of the induction strategy, and fine-tuning of conditions during both growth and bioconversion phases, resulted in further increases in product titers, reaching 72 g/L β-alanine and 6.15 g/L lycopene. This study offers valuable insights into the development of microbial co-production systems, highlighting the critical role of dynamic cofactor and redox balance management, as well as process optimization, in improving production efficiency.

## 1. Introduction

Synthetic biology and metabolic engineering have emerged as powerful platforms for constructing high-efficiency biosynthetic pathways, enabling the cost-effective production of valuable chemicals through rational pathway design and optimization. A fundamental principle in designing microbial cell factories lies in maximizing production efficiency while minimizing process costs. This objective is typically achieved through two synergistic strategies: (1) the utilization of low-cost feedstocks to reduce raw material expenses, and (2) the systematic optimization of metabolic networks to enhance pathway efficiency [1,2]. Critical to this optimization process is the reduction of metabolic resource expenditures, particularly through the minimization of carbon flux divergence, energy losses, and unnecessary consumption of reducing equivalents [3,4,5].

Fatty acids (FAs) represent an attractive alternative feedstock for bioproduction due to their highly reduced aliphatic hydrocarbon chains [6]. The catabolism of FAs through β-oxidation generates substantial quantities of acetyl-CoA, a central metabolic precursor, while simultaneously producing abundant reducing equivalents: nicotinamide adenine dinucleotide (NADH) and flavin adenine dinucleotide (FADH_2_). This dual output of both carbon skeletons and reducing power offers distinct advantages for biosynthetic applications, potentially enabling more sustainable and economically viable chemical production compared to conventional carbohydrate-based feedstocks. Notably, FA utilization aligns with circular economy principles as these substrates can be derived from low-cost sources, such as palm fatty acid distillate (PFAD), a palm oil-refining byproduct, and recycled waste cooking oils [7,8,9].

Our previous work established an efficient β-alanine biosynthesis platform using FA feedstocks [10]. As a versatile chemical with applications spanning pharmaceuticals, food additives, and advanced materials [11,12,13], β-alanine production was engineered through a pathway combining β-oxidation-derived acetyl-CoA with glyoxylate cycle-mediated β-alanine synthesis, achieving a yield of 1.24 g/g, significantly higher than that of the conventional glucose-based route. However, in our previous work, we observed that β-alanine productivity plateaued during the later phases of fermentation, with the underlying mechanisms yet to be fully elucidated [10]. We hypothesize that this stagnation results from inherent redox imbalances in the biosynthetic pathway. Excess NADH and FADH_2_ are produced during both fatty acid degradation and β-alanine synthesis, which could lead to a disruption in the balance of reducing power. As a result, the surplus reducing equivalents may inhibit fatty acid utilization and further β-alanine synthesis.

To address the imbalance in reducing power, an effective strategy is to couple a pathway that consumes reducing equivalents with one that generates them, achieved through the co-production of distinct products [14,15]. However, the effectiveness of this approach depends critically on the design and optimization of the specific metabolic pathways. More importantly, its feasibility when using FA-based feedstocks remains uncertain. In this study, we propose an innovative co-production strategy that synergistically couples β-alanine synthesis with lycopene production, both utilizing FAs as the primary feedstock. Lycopene, a powerful antioxidant with expanding applications in nutraceuticals and animal feed industries, requires substantial amounts of reducing cofactors and adenosine triphosphate (ATP) for its biosynthesis via the mevalonate (MVA) pathway [16,17,18,19]. Among carotenoids, lycopene has been the most extensively studied in metabolic engineering and is capable of achieving exceptionally high levels of accumulation [20]. It has also been successfully synthesized from fatty acid feedstocks via the MVA pathway, making it an ideal candidate for co-production with fatty acid-based raw materials [18]. Prior studies have shown that reducing power plays a crucial role in determining the efficiency of lycopene production [21]. The MVA pathway and the subsequent lycopene biosynthesis steps require large quantities of nicotinamide adenine dinucleotide phosphate (NADPH) and ATP, as well as the FADH_2_-coupled desaturation reaction [22]. As such, the lycopene biosynthetic pathway can effectively utilize excess reducing equivalents, thereby contributing to the mitigation of redox imbalance within the cell. Nevertheless, the distinct cellular localization of products—extracellular accumulation of β-alanine versus intracellular deposition of lycopene—enables spatial product segregation that inherently streamlines downstream purification processes. Through systematic metabolic engineering and comprehensive metabolite analysis, we (1) redesigned carbon flux distribution to optimally balance precursor supply between both pathways, (2) achieved unprecedented redox balance in FA-based production systems, and (3) demonstrated enhanced process economics through complementary product accumulation. This work establishes a new paradigm in green bioproduction, showcasing how redox-driven co-production strategies can simultaneously address metabolic challenges while improving overall process sustainability and economic viability.

## 2. Results

Rebalance the Redox State in β-Alanine-Producing Strain by introducing NADH oxidase (*nox*) and Downregulating NADH-generating Enzymes

**Figure 1:**
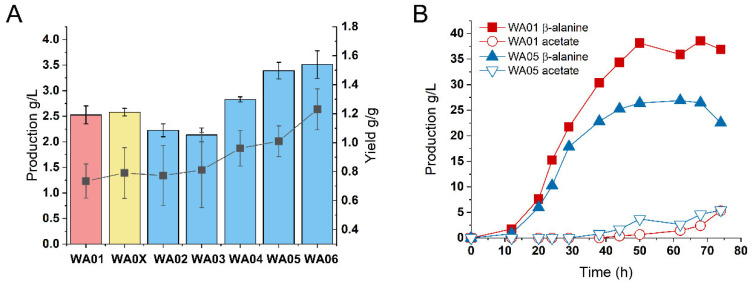
Comparative analysis of β-alanine production performance in different engineered strains. (**A**) Shake-flask level evaluation of β-alanine biosynthesis. The bar graph illustrates β-alanine titers achieved through whole-cell bioconversion using palmitic acid as a substrate over 18 h. The line graph represents the corresponding yield, calculated as the mass ratio of β-alanine produced to palmitic acid consumed. (**B**) Comparative fermentation profiles of strains WA01 and WA05 in 1-L bioreactors.

In previous studies, we constructed a pathway in *Escherichia coli* for the biosynthesis of β-alanine from FA as the substrate [10]. However, a major issue encountered was during the late stage of the fermentation bioconversion, where the production of β-alanine could not be further improved, and the bioconversion yield declined. To systematically investigate this bottleneck and clarify the metabolic profiles of both the β-alanine biosynthesis pathway and cell growth physiology, we employed strain WA01 (Table 1), which retains the key features for β-alanine production, in a one-step fermentation process. During the initial stage of fermentation, the strains first utilized glucose for biomass formation and induction of the recombinant pathway, followed by direct oil supplementation to support β-alanine production. The oil was replenished intermittently according to consumption. Monitoring revealed the complete stagnation of β-alanine accumulation after 50 h, concurrent with a decline in oil consumption efficiency and substantial acetate accumulation (5.33 g/L) (Figure 1B). These results identify a critical metabolic bottleneck occurring during the late-phase bioconversion.

**Figure 2:**
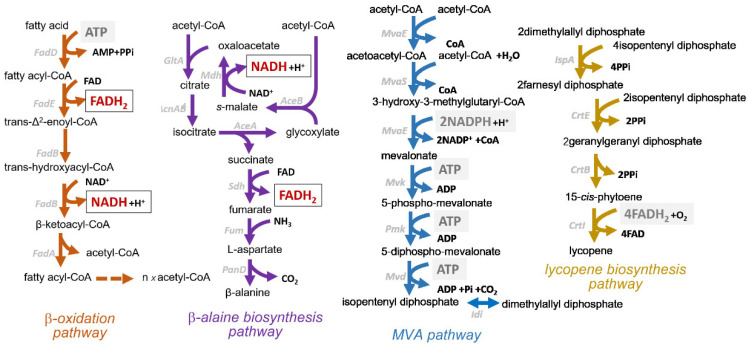
Reducing equivalent (NAD(P)H/FADH_2_) and ATP generation and consumption in engineered metabolic pathways.

We hypothesized that excessive NADH and FADH_2_ production during fatty acid β-oxidation (generating 1 NADH and 1 FADH_2_ per acetyl-CoA molecule) and β-alanine biosynthesis (producing 1 NADH and 1 FADH_2_ per molecule synthesized) (Figure 2), combined with NADH generation by TCA cycle enzymes, led to a severe redox imbalance that disrupted cellular metabolism. To alleviate this, two strategies were employed to reduce the accumulation of reduced cofactors and restore redox equilibrium.

We first introduced a heterologous NADH oxidase (*nox*) from *Streptococcus pyogenes* to oxidize excess NADH (strain WA0X) [23]. While this strategy marginally improved β-alanine titers in shake flask cultures (2.57 ± 0.07 g/L) (Figure 1A), scaling to 1L fermenters resulted in suppressed cell growth and a significant reduction in β-alanine production (19.43 g/L) in 72 h, with no alleviation of late-stage stagnation (Table 2). Second, to reduce the NADH-generating TCA enzyme’s activity, we modulated the TCA cycle flux by downregulating the expression of isocitrate dehydrogenase (*icd*). Our previous studies have shown that a complete block of *icd* impedes cell growth, though it may improve yield in flask cultures. Therefore, we chose to regulate icd expression through attenuation. Rare codon optimization was used to downregulate isocitrate dehydrogenase (*icd*), redirecting carbon flux from the NADH-generating TCA oxidation pathway to the glyoxylate shunt, thereby increasing precursor availability for β-alanine biosynthesis. Shake-flask experiments confirmed enhanced β-alanine production and yield in both the icd knockout strain (WA06) and icd-attenuated strains (WA02–WA05) (Figure 1A). However, during fermenter-scale cultivation, the top-performing flask strain among the icd-attenuated strains, WA05, reached a β-alanine titer of only 26.35 g/L at 50 h, followed by a decline in production and persistent acetate accumulation (5.43 g/L) (Figure 1B). Consistent with the previous findings, complete *icd* knockout severely impaired β-alanine synthesis and cell growth (Table 2). These results underscore the insufficiency of NADH-centric strategies alone to resolve redox-driven limitations.

### 2.1. Design and Rationale of a Dual-Product Synthesis Strategy for Redox Balancing

The conversion of FA to β-alanine generates an excess of NADH and FADH_2_, resulting in a significant redox imbalance. Stoichiometric analysis revealed that converting one molecule of palmitic acid to β-alanine generates substantial reducing power: (1) β-oxidation of a palmitate acid molecule yields 8 acetyl-CoA, 7 NADH, and 7 FADH_2_; (2) the subsequent condensation of two acetyl-CoA molecules into β-alanine produces 1 NADH; and (3) succinate-to-fumarate conversion in the TCA cycle contributes an additional FADH_2_ per cycle (Figure 2). This cumulative burden (2.75 NADH, 2.75 FADH_2_ per β-alanine molecule) overwhelms cellular redox homeostasis.

To address the redox imbalance caused by excessive NADH/FADH_2_ accumulation, we developed a co-production strategy to dynamically redistribute reducing equivalents. We sought to utilize excess reducing power to fuel cellular anabolism and growth. To further mitigate the redox imbalance, we engineered a biomass-linked synthesis pathway that directly consumes surplus reducing equivalents. This dual strategy ensures dynamic redox balance while coupling product synthesis to biomass formation. In the β-alanine-producing strain, we engineered a parallel lycopene biosynthesis pathway utilizing acetyl-CoA. Lycopene production through the mevalonate (MVA) and carotenoid biosynthesis pathways consumes NADPH (2 molecules per isopentenyl pyrophosphate), ATP (3 molecules per isopentenyl pyrophosphate), and FADH_2_ (4 molecules in phytoene desaturation) (Figure 2). Importantly, the intracellular accumulation of lycopene prevents competition with the extracellular secretion of β-alanine, allowing for spatial separation of the products.

### 2.2. Validation and Optimization of Dual-Pathway Strains for Enhanced Co-Production

**Figure 3:**
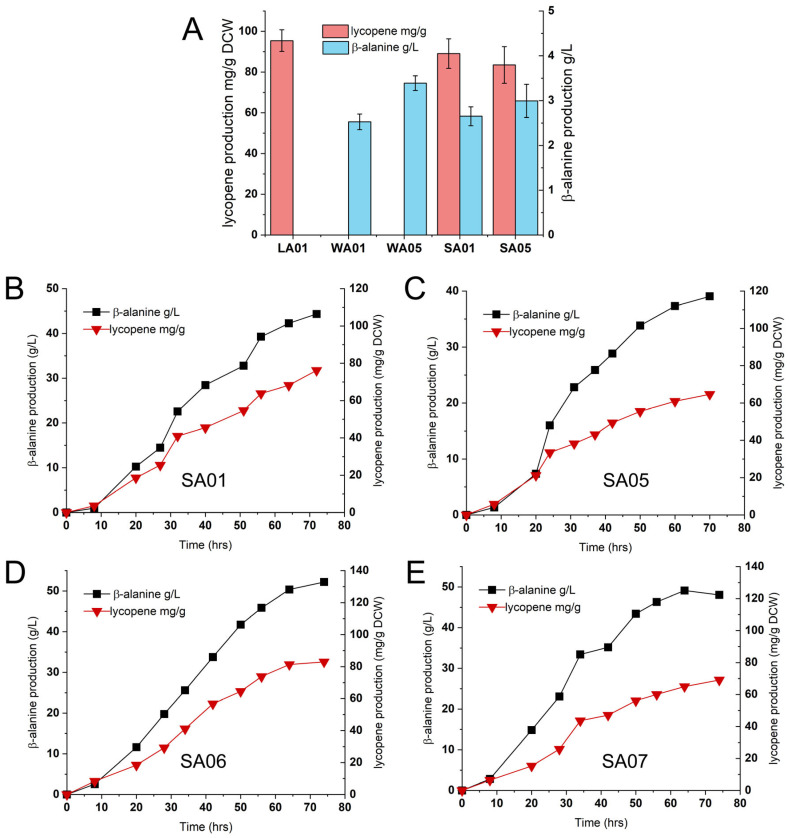
Comparative analysis of β-alanine production performance in dual-pathway strains. (**A**) Shake-flask level evaluation of lycopene and β-alanine production; (**B**) fermentation profiles of strains SA01 in 1-L bioreactor; (**C**) fermentation profiles of strains SA05 in 1-L bioreactor; (**D**) fermentation profiles of strains SA06 in 1-L bioreactor; (**E**) fermentation profiles of strains SA07 in 1-L bioreactor.

To validate the dual-pathway redox balancing strategy, the lycopene synthesis module was introduced into the two β-alanine-producing strains, WA01 and WA05. The MVA pathway genes were chromosomally integrated, while lycopene biosynthetic genes (*crtEBI*) were expressed from a low-copy-number vector, generating dual-pathway strains SA01and SA05. Shake-flask assays demonstrated that SA01 and SA05 retained lycopene production comparable to the lycopene-only control strain LA01 (89.1 ± 7.25, 83.52 ± 9.01, and 95.45 ± 5.32 mg/g DCW for SA01, SA05 and LA01, respectively), while β-alanine titers (2.65 ± 0.21 and 2.99 ± 0.37 g/L for SA01 and SA05, respectively) matched single-pathway parental strains (Figure 3A).

At the 1L fermenter scale, SA01 demonstrated significant advantages: both biomass and lycopene production increased substantially compared to LA01, with lycopene reaching 3.07 g/L. Additionally, β-alanine synthesis in SA01 continued through late fermentation, reaching 44.78 g/L at 70 h, representing a 19.8% increase over WA01. SA05 exhibited only a marginal improvement in β-alanine production (39.03 g/L), underscoring the critical role of TCA cycle flux in maintaining strain metabolism (Figure 3B; Table 2).

To enhance co-production efficiency, SA01 was further engineered: (1) *pntAB* overexpression and *sthA* deletion redirected NADH toward NADPH synthesis (strain SA06), aligning with MVA pathway cofactor demands; (2) The *crtEBI* genes were expressed using medium-copy-number vectors to enhance flux through the lycopene biosynthesis pathway, generating strain SA07. In fermenters (Figure 3D,E), SA06 exhibited a superior performance, with higher biomass (OD_600_: 135.9), lycopene production (3.61 g/L), and β-alanine production (52.78 g/L), all exceeding the levels observed in SA01. SA07 demonstrated no further improvement in strain performance, thereby establishing SA06 as the optimal strain. These results validate the dual-pathway strategy’s capacity to resolve redox constraints, sustain late-stage β-alanine synthesis, and enhance overall bioprocess efficiency.

### 2.3. Metabolite Analysis at Different Fermentation Stages of WA01 and SA06

**Figure 4:**
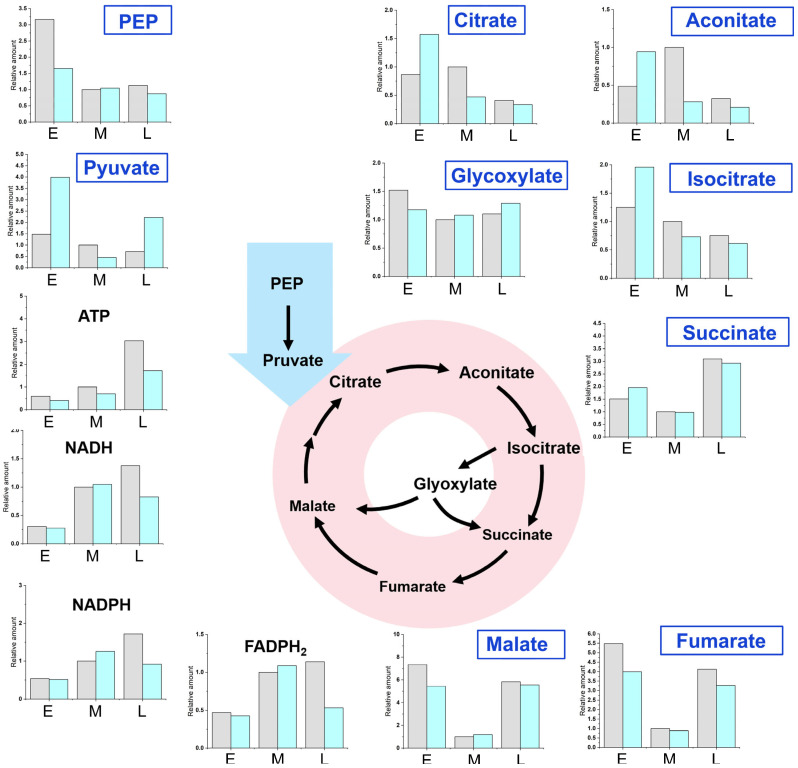
Metabolite analysis of strains WA01 (grey) and SA06 (blue) during fermentation. The stages of fermentation are represented as early (E, 20 h), middle (M, 45 h), and late (L, 60 h).

To elucidate the mechanism by which lycopene pathway integration promotes β-alanine synthesis and to investigate the synergistic effects of dual metabolic pathways, we conducted a comparative analysis of intracellular metabolite levels in WA01and SA06. These analyses were performed at three critical fermentation stages: the growth induction phase (20 h, E), middle fermentation phase (45 h, M), and late fermentation phase (60 h, L). Key intermediates from central metabolism and redox cofactors were monitored to uncover dynamic metabolic shifts (Figure 4).

During the growth induction period (E), intermediates of the central metabolic pathways (e.g., glycolysis, TCA cycle) were maintained at elevated levels in both strains. This suggests active substrate utilization and energy generation to support initial biomass accumulation. Upon transitioning to the fermentation phase (M), a marked reduction in central metabolic intermediates was observed, coinciding with the metabolic switch to fatty acid utilization as the primary carbon source. This shift indicates a reallocation of metabolic resources toward pathway-specific product synthesis. By the late fermentation stage (L), SA06 exhibited a pronounced decline in NADH and FADH_2_ levels compared to WA01. This significant reduction in redox cofactors demonstrates their efficient consumption in SA06, likely driven by the simultaneous activity of the β-alanine and lycopene synthesis pathways. The observed cofactor dynamics in SA06 provide critical evidence that the dual-pathway system enhances β-alanine production by effectively balancing redox cofactor regeneration and consumption. By the late stage of fermentation, SA06 also showed a significant increase in pyruvate level, a key central metabolite in cell growth, suggesting that metabolic flux was effectively redirected toward anabolic pathways.

### 2.4. Metabolic Bottleneck and Process Optimization Strategy

Metabolomic analysis revealed a substantial depletion of primary metabolic intermediates following oil supplementation during fermentation. This metabolic reprogramming likely reflects a survival-driven response, redirecting precursors to maintain essential cellular functions at the expense of product biosynthesis. Furthermore, the contrasting physiological demands of target products necessitated distinct fermentation paradigms: β-alanine production, a bulk chemical requiring carbon-efficient flux channeling, demanded biomass suppression, whereas lycopene synthesis as an intracellular metabolite relied on tight coupling with biomass accumulation.

To reconcile these competing objectives and mitigate carbon source transition stress, we developed an integrated fermentation strategy for SA06 strain. A stepwise carbon transition protocol was employed. Upon depletion of the initial glucose supply, the culture was supplemented with a glucose–oil mixture, wherein the oil-to-glucose ratio was progressively increased until induction was completed. Furthermore, inducer addition was delayed until the culture reached an OD_600_ of 110, alongside nutrient optimization during the initial oil supplementation phase: a 25% reduction in the soybean oil feed rate (1.35 g/L/h) and a doubling of MgSO_4_·7H_2_O supplementation (2 g/L). During the subsequent bioconversion phase, oxygen restriction (25% dissolved oxygen) and alkaline pH control (maintained at 7.6 via NH_4_OH) were employed to strategically modulate TCA cycle activity and oxidative phosphorylation, thereby balancing precursor availability with energy metabolism.

**Figure 5:**
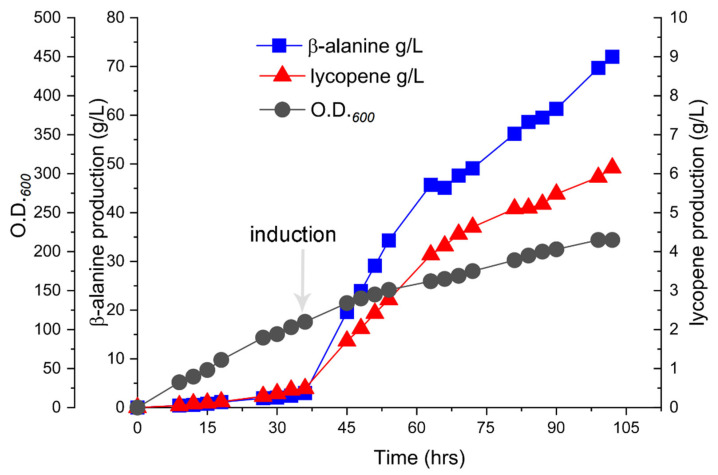
The optimization of the fermentation process for strain SA06.

This approach generated a 182% biomass surge (OD_600_ 215), elevating lycopene production to 6.15 g/L. Intriguingly, the β-alanine yield doubled to 72 g/L, underscoring the inherent metabolic competition between biomass-driven carotenoid synthesis and dedicated chemical bioproduction (Figure 5).

## 3. Discussion

Maintaining redox balance is a critical consideration in microbial strain pathway design for efficient chemical production [25,26]. However, developing effective strategies to achieve this balance remains a significant challenge. Previous studies have explored strategies to address reducing power imbalance through the co-production of different chemicals [27]. However, the effectiveness of such approaches largely depends on the specific design and optimization of the metabolic pathways involved. Additionally, industrial feasibility remains a key consideration. For example, when both products are secreted into the extracellular medium, downstream separation becomes more complex and must be carefully addressed [28].

In our study, we employed fatty acids, feedstocks with promising industrial applications, as the primary raw materials. This represents a significant departure from traditional designs based on glucose. Unlike glucose, the metabolism of fatty acids primarily generates NADH and FADH_2_ during β-oxidation to generate acetyl-CoA. As a result, achieving a balanced distribution of multiple cofactors, including NADPH, NADH, and FADH_2_, becomes a critical aspect of pathway design and optimization. This study demonstrates the successful co-production of lycopene and β-alanine through synergistic metabolic engineering and fermentation optimization. By integrating a heterologous lycopene biosynthetic pathway into a β-alanine-producing strain, we resolved redox imbalance caused by excess NADH/FADH_2_ generation, achieving coordinated synthesis of both products at 6.15 g/L (lycopene) and 72 g/L (β-alanine). The engineered strain showed a significantly reduced accumulation of reductive cofactors and enhanced production of both target compounds, confirming that pathway coupling is an effective strategy for cofactor management.

Process optimization through phased fermentation control further enhanced overall system performance. Unlike our previous study, which employed a staged whole-cell catalytic fermentation process, we adopted a one-step fermentation method in this work. In the dual-pathway strain, this approach not only maintained β-alanine production but also led to an increase in biomass, thereby further enhancing overall lycopene yield. Given that β-alanine production exceeded that of lycopene, we speculate that the additional reducing power generated may also play a crucial role in supporting normal cellular metabolism and biomass accumulation.

During the metabolic production of different compounds, fermentation strategies and metabolic engineering approaches must be tailored to the specific properties of each target product. For bulk chemicals such as β-alanine and L-aspartate, which are typically secreted extracellularly, the primary objective is to maximize yield from the raw material [10,29]. Although increasing biomass can enhance overall titer to some extent, it also elevates production costs and decreases yield efficiency relative to the feedstock. Therefore, a reduced biomass accumulation phase is generally preferred for β-alanine production, along with strategies to maximize enzyme expression per unit of biomass. In contrast, lycopene production depends not only on its accumulation per unit biomass but also correlates strongly with total biomass. Thus, increasing biomass is essential for improving lycopene yield. To address these differing production requirements in a co-production system, we developed a carbon source feeding strategy combined with delayed induction. This approach effectively supports the simultaneous accumulation of biomass and both target compounds, offering significant advantages for industrial-scale application. Furthermore, in dual-pathway strains, maintaining redox balance facilitates the coordinated production of β-alanine and lycopene, leading to a synergistic enhancement in overall productivity. In summary, this work underscores the pivotal role of cofactor ratio stabilization in complex pathway engineering and provides actionable insights for developing next-generation microbial platforms targeting high-value compound portfolios.

## 4. Materials and Methods

### 4.1. Chemicals and Reagents

DNA polymerase and Gibson kits were purchased from Vazyme (Nanjing, China). Restriction enzymes were acquired from New England Biolabs (Ipswich, MA, USA). For high-performance liquid chromatography (HPLC), β-alanine, lycopene, and other standards were obtained from Sigma-Aldrich (St. Louis, MO, USA). All other reagents were commercially available and of analytical grade.

### 4.2. Bacterial Strains and Cultivation Conditions

The strains used in this study are listed in Table 1. The *E. coli* BW25113 strain served as the chassis for strain engineering, while *E. coli* DH5α was used for general cloning. For plasmid construction, *E. coli* strains were grown at 37 °C for 16 h either in Luria–Bertani (LB) medium (10 g/L tryptone, 5 g/L yeast extract, 10 g/L NaCl) in a shaker at 220 rpm or on LB agar plates (1.5% agar, *w*/*v*), with antibiotics added as needed. For microbial cell preparation in flask experiments, overnight cultures were inoculated into LB medium with a 1% (*w*/*v*) inoculum and incubated at 30°C with constant shaking at 220 rpm for 16 h.

### 4.3. Genetic Manipulations

DNA manipulations were conducted using standard protocols. Target gene deletion, integration, and replacement were performed with a CRISPR-Cas9-mediated gene editing system [30]. The CRISPR procedure was performed following the method recommended in the literature. The targeting fragment contained 500 bp homology arms flanking the upstream and downstream regions. The sgRNA sequences, primers, homology arms, and exogenous DNA sequences used in this study are listed in Supplementary Tables S1 and S2. L-Arabinose (2.0 g/L) was added to induce CRISPR/Cas9 system during gene deletion and integration. The plasmids used in this study are listed in Table 1. Mutant strains were confirmed by PCR and verified by gene sequencing (RuiBiotech Co., Beijing, China).

### 4.4. Analytical Methods

Cell growth was monitored by measuring optical density at 600 nm (Ultrospec 3000, Amersham Pharmacia Biotech, Piscataway, NJ, USA). For β-alanine quantification, samples were derivatized with dinitrofluorobenzene (DNFB) in 0.1 M NaHCO_3_ at 60 °C for 1 h, quenched with 0.1% formic acid, and filtered (0.22 μm PES membrane) before HPLC analysis. Separation used an Ascentis Express C18 column (4.6 × 250 mm, 2.7 μm, Merck KGaA, Darmstadt, Germany) at 40 °C with 0.1% formic acid/acetonitrile (65:35) mobile phase at 0.5 mL/min, detecting at 360 nm. Standard curve was determined using standard β-alanine (Supplementary Figure S1). Lycopene was extracted from cells using methanol:ethyl acetate (1:1) at 4 °C for 3–4 h. After centrifugation and filtration (0.22 μm), analysis was performed by HPLC with a Hypersil BDS C8 column (150 × 4.6 mm, 3 μm, Thermo Fisher Scientific, Waltham, MA, USA) at 30 °C, using methanol (A) and methanol/0.1 M ammonium acetate (7:3) (B) as mobile phases with gradient elution (1 mL/min), detecting at 450 nm. Standard curve was determined using standard lycopene (Supplementary Figure S2). To confirm lycopene production, the fraction corresponding to the HPLC peak was collected, lyophilized, and analyzed by ^1^H and ^13^C NMR spectroscopy at 25 °C using a Bruker Avance III HD 500 MHz spectrometer (Bruker BioSpin AG, Fällanden, Switzerland). NMR analysis verified that the substances corresponding to the HPLC peak were pure lycopene and structurally accurate (Supplementary Figure S3). Organic acids were analyzed by HPLC with an Aminex HPX-87H column (300 × 7.8 mm, Bio-Rad Laboratories, Hercules, CA, USA) using 5 mM H_2_SO_4_ mobile phase at 0.6 mL/min (55 °C). Fatty acids were converted to methyl esters (10% H_2_SO_4_ in methanol, 60 °C, 20 min), extracted with hexane, and analyzed by GC-FID with an HP-88 column (60 m × 0.25 mm, 0.2 μm, Agilent Technologies, Santa Clara, CA, USA) using the following temperature program: 150 °C (5 min), 3 °C/min to 170 °C (5 min), then 3 °C/min to 210 °C (5 min). The analysis of intracellular metabolites was conducted according to the methods described in the literature [10]. All analyses were performed in triplicate.

### 4.5. Flask and Fed-Batch Bioconversion

A monoclonal colony was picked from LB agar plates containing antibiotics and inoculated into 5 mL of liquid LB medium with antibiotics to prepare the seed culture. In shake-flask experiments, the culture was initially grown in LB medium to an OD_600_ of 1.0. Then, arabinose was added to a final concentration of 0.2%, and induction was carried out at 30 °C for 16 h. The cells were then collected and transferred to M9 medium containing 0.5% palmitic acid for whole-cell bioconversion for 18 h. During the 1L fermentation process, the cells were first inoculated into CD medium containing glucose (10 g/L glucose, 14 g/L KH_2_PO_4_, 4 g/L (NH4)_2_HPO_4_, and 1.8 g/L citric acid monohydrate), and induction was initiated by adding 0.2% arabinose. After the initial glucose was consumed, induction was continued for a certain period, and soybean oil containing esterases was added for β-alanine and lycopene production. Experimental data from shake-flask cultures are presented as mean values derived from three independent biological replicates. For the 1 L scale fermentations, a representative dataset from three separate fermentation batches was selected based on consistent metabolic profiles and production trends observed throughout the fermentation timeline under identical cultivation conditions.

## Figures and Tables

**Table 1:** Strains used in this study.

***E. coli*** Strains	Genotypes	Reference
BW25113	F^−^ DE(*ara*D-*ara*B)567 *lac*Z4787(del)::*rrn*B-3 LAM^−^ *rph*-1 DE(*rha*D-*rha*B)568 *hsd*R514	CGSC ^#^
DH5α	F^−^ φ80*lac*ZΔM15 Δ(*lac*ZYA-*arg*F)U169 *rec*A1 *end*A1 *hsd*R17(r_K_^−^, m_K_^+^) *pho*A *sup*E44 λ^−^*thi*-1 *gyr*A96 *rel*A1	CGSC ^#^
WA01	BW25113, Δ*fad*R, P_CPA1_-*fad*D, P_119_-*fad*L, Δ*icl*R::P_119_-*Bspan*D, Δ*asp*C::P_119_-*glc*B-RBS-*ace*A, P_119_-*btu*E, P_119_-*gor*, P_CPA1_-*aspA*, pXB1k-*TcpanD* (p15A ori, Kan^R^) * [10]	This study
WA0X	WA01, pSB1s-*Bsnox* (pSC101 ori, Str^R^) * [23]	This study
WA02	WA01, GTG-*icd*	This study
WA03	WA01, ATGAGG-*icd*	This study
WA04	WA01, ATG(AGG)_2_-*icd*	This study
WA05	WA01, ATG(AGG)_3_-*icd*	This study
WA06	WA01, Δ*icd*	This study
LA01	BW25113, ΔlpxM::P_araBAD_-*mva*S-*mva*E-*mvk*-P_araBAD_*-pmk-mvd-idi*, pSB1s-*crt*EBI (pSC101 ori, Str^R^) * [18]	This study
SA01	WA01, Δ*lpx*M::P_araBAD_-*mva*S-*mva*E-*mvk*-P_araBAD_*-pmk-mvd-idi*, pSB1s-*crt*EBI	This study
SA05	WA05, Δ*lpx*M::P_araBAD_-*mva*S-*mva*E-*mvk*-P_araBAD_*-pmk-mvd-idi*, pSB1s-*crt*EBI	This study
SA06	SA01, Δ*sth*A, P_119_-*ptn*AB	This study
SA07	SA06, pSB1s-*crt*EBI > pMB1s-*crt*EBI (MBI ori, Str^R^) * [24]	This study

* Details of the promoters, MVA pathway genes, lycopene biosynthesis genes, β-alanine biosynthesis genes, and plasmid vectors used in this study are described in references [10,18,23,24]. ^#^ The *E. coli* Genetic Resource Center.

**Table 2:** Strain performance in 1 L fermenter.

Strains	Lycopene Production (72 h)		β-Alanine Production		O.D._600_
	mg/g DCW	g/L	50–51 h	70–72 h	
LA10	56.4	1.76	0	0	97.5
WA01	0	0	38.13	36.87	86.3
WA0X	0	0	16.76	19.43	68.0
WA05	0	0	26.38	22.53	64.1
WA06	0	0	11.22	7.49	46.2
SA01	76.20	3.07	36.79	44.34	125.6
SA05	64.70	2.17	33.83	39.06	104.5
SA06	83.01	3.61	41.72	52.21	135.9
SA07	69.08	2.92	43.41	48.05	131.6

## Data Availability

Data is contained within the article and Supplementary Materials.

## Supplementary Materials

The following supporting information can be downloaded at: https://www.mdpi.com/article/10.3390/ijms26146727/s1.

## Abbreviations

FA(s): fatty acid(s); CoA: Coenzyme A; NADH: nicotinamide adenine dinucleotide; FADH2: flavin adenine dinucleotide; PFAD: palm fatty acid distillate; ATP: adenosine triphosphate; MVA: mevalonate; HPLC: high-performance liquid chromatography; TCA: tricarboxylic acid; NADPH: nicotinamide adenine dinucleotide phosphate; CRISPRs: clustered regularly interspaced short palindromic repeats; nox: NADH oxidase; icd: isocitrate dehydrogenase.
