# Supplementary material for: Synergistic Production of Lycopene and β-Alanine Through Engineered Redox Balancing in Escherichia coli
Source: Int J Mol Sci. 2025 Jul 14;26(14):6727. doi: 10.3390/ijms26146727 (PMC12294996; doi:10.3390/ijms26146727)
Supplement: Supplementary file 1 [file ijms-26-06727-s001.zip › ijms-3652301-supplementary.pdf]

To perform the CRISPR operation, primer pairs X-N20-F/R were used to amplify the target sequence from the pTargetF plasmid via PCR. The PCR product was then treated with DpnI and transformed into E. coli DH5α to generate a pTarget derivative plasmid targeting specific genomic sites (see Supplementary Table 1 for details). The targeting fragments contained upstream and downstream homology arms (200–500 bp) corresponding to the target sites. The sequences of the homology arms, along with the inserted fragments between them, are provided in Supplementary Table 2. Primer pairs X-F/R, used for amplifying the targeting fragments, are also listed in Supplementary Table 1.

Supplementary Table1. Primers used in this study

Purpose	Primers for Amplification of Target Fragments	Primers for Construction of Target-Specific pTarget Derivatives
generating strains WA02-05	icd-F:<br>5′-tctaaaagaagttttttgcatggtattttca-3′<br>icd-R:<br>5′-aggcagccagacgtcctgaccataaacctg-3′	icd-N20-F:<br>5′-ggaaagtaaagtagttgttccgggttttagagctagaaatagcaag-3′<br>icd-N20-R:<br>5′-ccggaacaactactttactttccactagtattataccta ggactgagc-3′
generating strain WA06	icd-F:<br>5′-Tctaaaagaagttttttgcatggtattttca-3′<br>icd2-R:<br>5′-taacagtgaatacataactaactttgggg-3′	icd-N20-F:<br>5′-ggaaagtaaagtagttgttccgggttttagagctagaaatagcaag-3′<br>icd-N20-R:<br>5′-ccggaacaactactttactttccactagtattatacctaggactgagc-3′
generating strains LA01, SA01, and SA05	lpxM-F:<br>5′-agaggtgttgatttcgccatgccgcagggtac-3′<br>lpxM-R:<br>5′-cgacaatgtggaagaagctattgccatgtcagc-3′	lpxM-N20-F:<br>5′-atgatcatacgattgcgcgggttttagagctagaaatagcaag-3′<br>lpxM-N20-R:<br>5′-ccgcgcaatcgtatgatcatactagtattatacctaggactgagc-3′
generating strain SA06	sthA-F:<br>5′-ggcactataccagagaatgaacatactgga-3′<br>sthA-F:<br>5′-gtggcggcaggtagcgggatcactttac-3′	sthA-N20-F:<br>5′-gatgccatagtaataggttccgggttttagagctagaaatagcaag-3′<br>sthA-N20-R:<br>5′-ccggaacctattactatggcatcactagtattatacctaggactgagc-3′
generating strain SA06	pntAB-F:<br>5′-ccaggtactggtattgttattaacgagaaa-3′<br>pntAB-R:<br>5′-gcgttgtcccaggattcagtaacgcaattt-3′	pntAB-N20-F:<br>5′-gcttgtgtggctcctgacacagggttttagagctagaaatagcaag-3′<br>pntAB-N20-R:<br>5′-cctgtgtcaggagccacacaagcactagtattatacctaggactgagc-3′

Supplementary Table2. Sequences of the homology arms and inserted fragments

	upstream homology arms (5′-3′)	downstream homology arms (5′-3′)
icd target in WA02-05	tctaaaagaagtttttttgcatggtattttcagagattatgaattgcc<br>gcattatagcctaataacgcgcatctttcatgacggcaaacaata<br>gggtagtattgacaagccaattacaaatcattaacaaaaaaattgc<br>tctaaagcatccgtatcgcaggacgcaaacgcatatgcaacgtg<br>gtggcagacgagcaaaccagtagcgctcgaaggagaggtga	cacaaggcaagaagatcaccctgcaaaacggcaaactcaacgttcc<br>tgaaaatccgattatcccttacattgaaggtgatggaatcggtgtag<br>atgtaaccccagccatgctgaaagtggtcgacgctgcagtcgagaa<br>agcctataaaggcgagcgtaaaatctcctggatggaaatttacacc<br>ggtgaaaaatccacacaggtttatggtcaggacgtctggctgcct
icd target in WA06	tctaaaagaagtttttttgcatggtattttcagagattatgaattgcc<br>gcattatagcctaataacgcgcatctttcatgacggcaaacaata<br>gggtagtattgacaagccaattacaaatcattaacaaaaaaattgc<br>tctaaagcatccgtatcgcaggacgcaaacgcatatgcaacgtg<br>gtggcagacgagcaaaccagtagcgctcgaaggagaggtga	tgccgtagtttgttaaatttattaacgggagcgtaacgctcccgttgtt<br>ttttgttaggctgctaacggttatcaaaattttatcaaaaaaagttatca<br>aaaccccctcggtagttttggggtaggctggccggtcaggtggtagtt<br>ctactactagtctcccacatagatattccttagctttttattattgctggc<br>ggacgctcgttaatatttaaggtcttcattgattaagacatccccaaa<br>gttagttatgtattcactgtta
lpxM target	agaggtgttgatttcgccatgccgcagggtacgccagtgctttca<br>gtgggtgacggtgaagtggtggttgccaaacgcagtggcgcag<br>caggttattatgtggctattcgtcatggtcgcagctacaccacgcg<br>ttatatgcacttgcgcaagattctggtgaaaccgggacagaagg<br>tgaaacgtggcgaccgtatcgcgctttccggtaataccggacgtt<br>caaccgggccgcatctgcactatgaagtatggataaaccagcag<br>gccgtaaacccgctgacggcaaaactgccgcgtaccgaagggc<br>tgaccggctccgatcgtcgcgaattcctggcgcaggccaaagag<br>attgtgccgcagctacggtttgattaattaacatccattcgcagcc<br>ggtacgcagtcagtaccggctttttttatttggtgcggggcaagtt<br>gcgccgctacactatcaccagattgatttttgccttatccgaaactg<br>gaaaagc	aaaagcctctcgcgaggagaggccttcgcctgatgataagttcaag<br>tttgcttcagaatattcgaaatctgttgaactatcattgaactgtaggc<br>cggatgtggcgttttcgccgcatccggcaacgtacttactctaccgtt<br>aaaatacgcgtggtattagtagaacccacggtactcatcacgtcgcc<br>ctgggtgacaatcaccaggtcaccagacatcaagtaacctttatcgc<br>gcagcagattaaccgcttcgctggcagctgctacgccgtcattagcg<br>ctatcaaagtgcaccggcgtaacgccacgatagagagcagtcagg<br>ttcagcgtacgttcatggcgcgacatggcgaaaattggcagaccag<br>agctgatacgggaggtcatcagcgcggtacgacccgattcggtcat<br>ggtgatgatcgccgtaacgcctttcaggtggttagctgcgtacattg<br>ctgacatggcaatagcttcttccacattgtcg
sthA target	ggcactataccagagaatgaacatactggattgctccagtattgt<br>gaatgaacggtaacgcaataaaacatatttactgcaattctttggc<br>cggttcttttacgtacagcggaaacctgccgcttaaacggagagt<br>atcgtcgataaaaatccaataaaacgtcagggcaaaagtaagaa<br>acagacaaagcaaaggccgctcaggatatagccagataaatga<br>cggggatcaattggcttacccgcgataaaatgttaccattctgttg<br>cttttatgtataagaacaggtaagccctacc	actttatcgaaatggccatccattcttgcgcggatggcctctgccagc<br>tgctcatagcggctgcgcagcggtgagccaggacgataaaccagg<br>ccaatagtgcggcgtggttccggcttaatgcacggcagataaacaa<br>ccccatcgcgtttgcgctccggcggcacagccagcgctggcagtaa<br>agtgatcccgctacctgccgccac
pntAB target	ccaggtactggtattgttattaacgagaaacgtggctgattattgc<br>atttaaacggtgtaactgtctgcgtcatttttcatatcacattccttaa<br>gccaattttaatcctgctcaaatgaccgtctatgcttaaaaaacag<br>ccgtatcagcatcattactactgaagcaactgaattgtataagtta<br>atttaatgttaagtagtgattcgtgccggggcgatgtctcgttttac<br>ccgaccgtcgaagacaattatcagtctttatccggcgttctaaggt<br>gtttatcccactatcacggctgaatcgttaatattttgcgagttcac<br>gccgaaatactgatttttggcgctagatca	atgcgaattggcataccaagagaacggttaaccaatgaaacccgtg<br>ttgcagcaacgccaaaaacagtggaacagctgctgaaactgggttt<br>taccgtcgcggtagagagcggcgcgggtcaactggcaagttttga<br>cgataaagcgtttgtgcaagcgggcgctgaaattgtagaagggaa<br>tagcgtctggcagtcagagatcattctgaaggtcaatgcgccgttag<br>atgatgaaattgcgttactgaatcctgggacaacgc
inserted fragment targeting icd in	5′-atggaaagtaaagtagttgttccgg-3′	

WA02	
inserted fragment targeting icd in WA03	5′-atggaaagtaaagtagttgttccgg-3′
inserted fragment targeting icd in WA04	5′-atggaaagtaaagtagttgttccgg-3′
inserted fragment targeting icd in WA05	5′-atggaaagtaaagtagttgttccgg-3′
inserted fragment targeting lpxM in LA01, SA01, and SA05	ParaBAD-mvaS-mvaE-mvk-ParaBAD-pmk-mvd-idi [18]
inserted fragment targeting pntAB in SA06	5′-atggcttgtcatgcttaattgacagctagctcagtcctaggtataatgctagcagggagaccacaacggtttccctctacaaataattttgtttaactttcgcgcgcgtaacaggaggaattaacc-3′

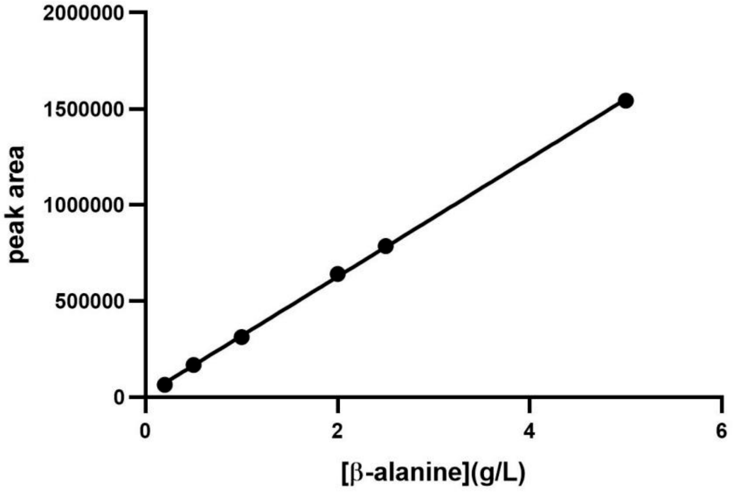

Supplementary Fig1. The standard curve for β-alanine was established by HPLC using a standard solution of β-alanine (Sigma-Aldrich). The resulting calibration equation was y = 307746x + 11127, R^2=0.9998.

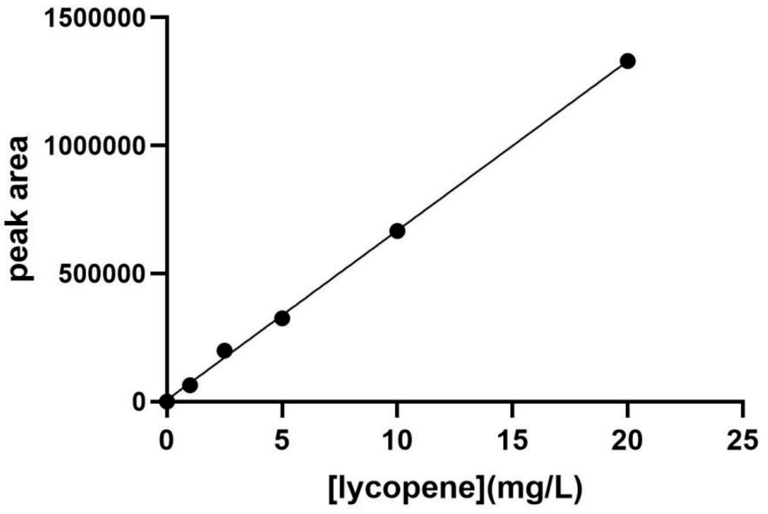

Supplementary Fig2. The standard curve for lycopene was established by HPLC using a standard solution of lycopene (Sigma-Aldrich). The resulting calibration equation was y = 66171x + 6617, R^2=0.999.

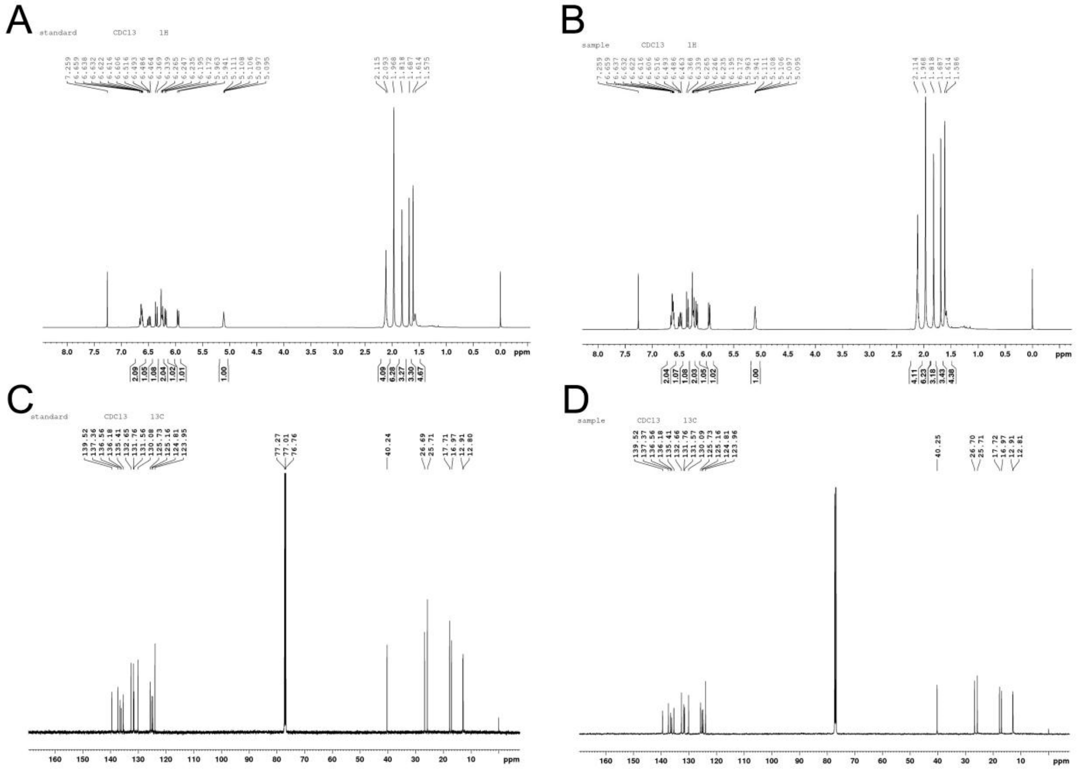

Supplementary Fig3. NMR spectrum corresponding to the lycopene HPLC peak. A. ^{1}H NMR spectrum of lycopene standard; B. ^{1}H NMR spectrum of sample; C. ^{13}C NMR spectrum of lycopene standard; D. ^{13}C NMR spectrum of sample.
